# Thermal Stability of Phase-Separated Domains in Multicomponent Lipid Membranes with Local Anesthetics

**DOI:** 10.3390/membranes7030033

**Published:** 2017-06-29

**Authors:** Ko Sugahara, Naofumi Shimokawa, Masahiro Takagi

**Affiliations:** School of Materials Science, Japan Advanced Institute of Science and Technology, 1-1 Asahidai, Nomi, Ishikawa 923-1292, Japan; koblack0083@jaist.ac.jp (K.S.); nshimo@jaist.ac.jp (N.S.)

**Keywords:** local anesthetics, liposome, phase separation, line tension

## Abstract

The functional mechanisms of local anesthetics (LAs) have not yet been fully explained, despite their importance in modern medicine. Recently, an indirect interaction between channel proteins and LAs was proposed as follows: LAs alter the physical properties of lipid membranes, thus affecting the channel proteins. To examine this hypothesis, we investigated changes in thermal stability in lipid membranes consisting of dioleoylphosphocholine, dipalmitoylphosphocholine, and cholesterol by adding the LAs, lidocaine and tetracaine. The miscibility temperature of liquid-ordered (L_o_) and liquid-disordered (L_d_) phase separation was lowered, whereas that of phase separation between solid-ordered (S_o_) and L_d_ phases was unchanged by LAs. Furthermore, we measured the line tension at the L_o_/L_d_ interface from domain boundary fluctuation and found that it was significantly decreased by LAs. Finally, differential scanning calorimetry (DSC) revealed a change in the lipid main transition temperature on the addition of LAs. Based on the DSC measurements, we considered that LAs are partitioned into two coexisting phases.

## 1. Introduction

Local anesthetics (LAs) are essential drugs, especially in modern surgical medicine, and are frequently used to suppress pain. The mechanism behind the suppression of pain signals by LAs is believed to result from the deactivation of sodium ion channel proteins and the inhibition of the action potentials of neural cell membranes [1,2,3]. Molecular simulations have partly explained the inhibition of ion channels as direct interactions between LAs and the binding sites on the proteins [4,5]. These studies have demonstrated the closure behavior of cation-gate structures that accompanies conformational change. However, the direct interaction hypothesis does not adequately explain the deactivation of ion channels by LAs.

Recent studies have suggested indirect interactions between the channels and LAs [6,7]. Since most LA molecules have large hydrophobic parts in their structures, LAs may interact with the hydrophobic region and alter the physical properties of the biomembranes. As a result, the channel proteins in biomembranes would be indirectly influenced by LAs through changes in the physical properties of the membranes. Some groups have focused on the hydrophobicity of LAs [8] and examined the interactions between phospholipid membranes and LAs using nuclear magnetic resonance (NMR) [9,10]. In addition, it has been reported that the structures of membrane proteins are influenced by the bending rigidity of lipid membranes [11]. Some studies have found that the membrane tension mediated by a mechanical stimulus affects the function of ion channels [12]. As shown in these cases, modulation of the function of membrane proteins via changes in the physical properties of the membrane, such as bending rigidity and membrane tension, is central to understanding the function and behavior of membrane proteins.

The importance of indirect interactions between channel proteins and LAs has attracted much attention, and the interactions between biomembranes and LAs are thus worthy of investigation. Biomembranes, which are mainly composed of a phospholipid bilayer structure, are known to be involved in signal transduction mechanisms, such as vesicle formation [13]. Furthermore, it is believed that compositional heterogeneity emerges spontaneously in biomembranes. These heterogeneous structures are known as “lipid rafts” [14,15]. The lipid raft is a specific region containing a large amount of cholesterol (Chol) and saturated phospholipids, as well as some functional proteins including ion channel proteins and membrane receptors. Therefore, lipid rafts are thought to be important platforms for signal transduction. Additionally, sodium ion channel proteins, which can be affected by LAs, are also known to exist in lipid rafts [16]. Hence, it is important to clarify the correlation between lipid rafts and biosignal transductions such as those that occur in anesthesia.

Due to the complexities of biomembranes, however, it is difficult to investigate the stability of the lipid raft by adding LAs. Thus, to reveal the interactions between lipid rafts and additive molecules such as LAs, liposomes—regarded as model biomembranes—are widely used. In particular, typical multicomponent lipid membranes consisting of unsaturated lipids, saturated lipids, and Chol exhibit phase separation between the unsaturated lipid-rich liquid-disordered (L_d_) phase and the saturated lipid/Chol-rich liquid-ordered (L_o_) phase [17,18,19,20,21,22]. Since the L_o_ phase is composed of saturated lipids and Chol, it is considered a suitable model of the raft region. The adsorption of LAs onto solid-ordered (S_o_) phase, consisting of saturated lipids and L_o_ phases, has been investigated by quartz crystal microbalance with dissipation [23,24]. Furthermore, we have observed the suppression of phase separation on raft-mimetic liposomes composed of unsaturated and saturated lipids and Chol via microscopy. Since the fluidity of the L_d_ phase decreases on the addition of LAs and the fluidity gap between the L_o_ and L_d_ phases reduces, these events result in the suppression of phase separation [25]. Conversely, in the case of binary lipid mixtures consisting of unsaturated and saturated lipids, the fluidity difference between the S_o_ and L_d_ phases is not adequately reduced. Consequently, phase separation between S_o_ and L_d_ phases is not suppressed by LAs [25].

Furthermore, from a physiological aspect, anesthesia via LAs can be enhanced by dosing at higher temperatures [26]. Therefore, it is also important to discuss the thermal stability of the L_o_ phase in the presence of LAs. Differential scanning calorimetry (DSC) measurements have revealed that the thermal stabilities of the S_o_ and L_o_ phases are decreased by LAs [23,24,27]. Gray et al. revealed that lowering the miscibility temperature, defined as the temperature for the transition between phase separation and the homogeneous phase, via the addition of liquid general anesthetics using giant plasma membrane vesicles isolated from living cells, correlates with the strength of general anesthetics [28]. Although the effects of LAs depend on the presence of Chol, the relationship between LAs and Chol is not fully understood.

In this study, we investigated the thermal stability of the phase-separated structures in biomimetic lipid membranes containing LAs. First, the miscibility temperatures in unsaturated lipids, dioleoylphosphocholine (DOPC)/saturated lipids, dipalmitoylphosphocholine (DPPC)/LAs, and DOPC/DPPC/Chol/LAs were measured by fluorescence microscopy. Next, we measured the line tension at the L_o_/L_d_ interface from the fluctuation of the domain boundary in DOPC/DPPC/Chol/LAs to examine the stability of the domain. Finally, DSC measurements were used to clarify the lipid transition temperatures in LA-containing lipid membranes. From the results of the DSC experiments, it was considered that LAs are partitioned as coexisting phases. Moreover, we discussed the influence of LAs on the thermal stability of phase-separated domains in multicomponent lipid membranes.

## 2. Materials and Methods

### 2.1. Materials

**Figure 1:**
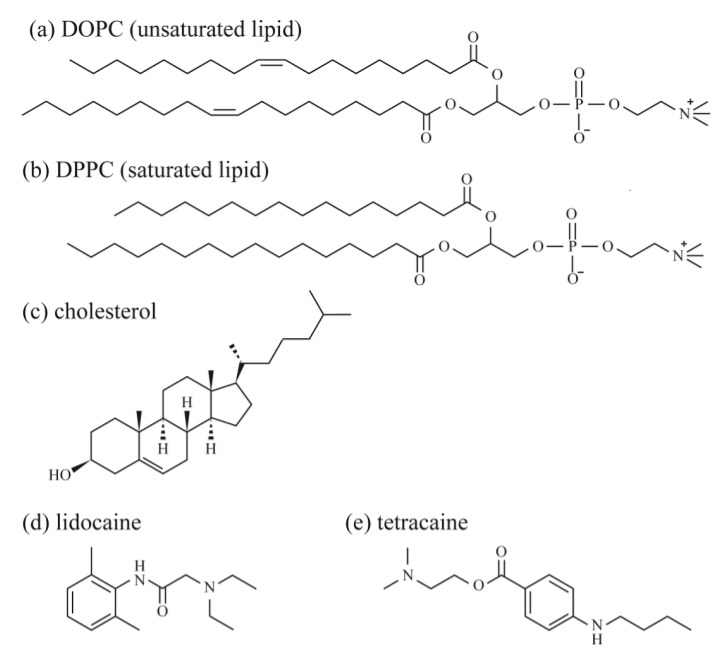
Chemical structures of (**a**) DOPC; (**b**) DPPC; (**c**) Chol; (**d**) lidocaine; and (**e**) tetracaine.

Unsaturated lipids, 1,2-dioleoyl-*sn*-glycero-3-phosphocholine (DOPC), saturated lipids, 1,2-dipalmitoyl-*sn*-glycero-3-phosphocholine (DPPC), and Chol were purchased from Avanti Polar Lipids (Alabaster, AL, USA). Fluorescent probes, Rhodamine B 1,2-dihexadecanoyl-*sn*-glycero-3 phosphoethanolamine, and triethylammonium salt (Rhod-DHPE) were purchased from Thermo Fisher Scientific (Waltham, MA, USA). Rhod-DHPE molecules are mainly located in the DOPC-rich L_d_ phase. The LAs 2-(diethylamino)-*N*-(2,6-dimethylphenyl)acetamide (lidocaine) and 2-(dimethylamino)ethyl 4-(butylamino)benzoate (tetracaine) were purchased from Nacalai Tesque (Kyoto, Japan) and Tokyo Chemical Industry (Tokyo, Japan), respectively. Ultrapure water (specific resistance ≥18 MΩ) was obtained using a Millipore Milli-Q purification system (Merck Millipore, Billerica, MA, USA). The chemical structures of the lipids and LAs are shown in Figure 1.

### 2.2. Experimental Methods

#### 2.2.1. Microscopic Observation of Phase-Separated Structure on Liposomes

Liposomes for microscopic observation were prepared by the natural swelling method. Lipids (DOPC, DPPC, Chol), LAs (lidocaine, tetracaine), and the fluorescent probe (Rhod-DHPE) were dissolved in chloroform, at concentrations of 2, 0.5 and 0.1 mM, respectively. The stock solutions were transferred into glass test tubes and mixed to the desired compositions. The organic solvent was evaporated under a flow of nitrogen gas, and the lipids were further dried under vacuum for least 3 h to form thin lipid films. The films were then hydrated overnight with Milli-Q water at 55 °C to produce unilamellar liposomes. The final concentrations of lipids and LAs were 0.2 mM, and that of Rhod-DHPE was 1 μM.

To observe the effects of LAs on phase separation by fluorescent microscopy, we added LAs to DOPC/DPPC lipid mixtures with a DOPC:DPPC ratio of 1:1, and to the DOPC/DPPC/Chol lipid mixtures with a DOPC:DPPC:Chol ratio of 2:2:1. Therefore, the compositions examined were DOPC/DPPC/LAs 50:50:0, 45:45:10; and 40:40:20, and DOPC/DPPC/Chol/LAs 40:40:20:0, 36:36:18:10, and 32:32:16:20. The sample temperature was controlled using a microscope stage (MATS-555MORA-BU, Tokai hit, Shizuoka, Japan) at 20–34 °C.

We counted 30 liposomes in each composition at each temperature, and plotted the fraction of phase-separated liposomes. Based on the experimental plots, we calculated the miscibility temperature (*T*_mix_) for the multicomponent lipid membranes. The miscibility temperature is defined as the temperature at which the fraction of phase-separated liposome reaches 50%. To obtain the miscibility temperature, the experimental results were fit with the sigmoidal Boltzmann function:

(1) $$P=\frac{1}{1+\exp\left[\left(T-T_{\mathrm{mix}}\right)/dt\right]}$$

where *P* is the fraction of phase-separated liposomes, *T* is the temperature, *T*_mix_ is the miscibility temperature, and *dt* is the slope of the sigmoidal curve.

#### 2.2.2. Line Tension Measurement by Flicker Spectroscopy of Domain Boundary Fluctuation

We obtained the line tension from the domain boundary fluctuation of a liquid domain [29,30]. Domains were imaged for 1 s at 30 frames/s. Traces of the domain boundary were obtained from binarized images using ImageJ (Ver. 1.49, National Institutes of Health, Bethesda, MD, USA). The radius from the center of mass of domain *r* as a function of the polar angle ψ was represented in terms of a Fourier series expansion:

(2) $$r\left(\psi \right)=r_{\mathrm{av}}\;\left[1+a_{0}+\overset{\infty }{\underset{k=1}{\sum }}a_{k}\cos\left(k\psi \right)+\overset{\infty }{\underset{k=1}{\sum }}b_{k}\sin\left(k\psi \right)\right]$$

where *r*_av_ is the average domain radius, *k* is the mode number, and *a_k_*, *b_k_* are the Fourier coefficients. The excess free energy arises due to the fluctuation and is expressed as:

(3) $$\Delta F≃\frac{\pi r_{\mathrm{av}}}{2}\gamma \overset{\infty }{\underset{k=2}{\sum }}\left(k^{2}-1\right)\left(a_{k}^{2}+b_{k}^{2}\right)$$

where γ is the line tension. The free energy for each independent mode becomes *k*_B_*T* from the generalized equipartition theorem, where *k*_B_ is the Boltzmann constant. Therefore, we obtained the equation:

(4) $$\langle a_{k}^{2}\rangle +\langle b_{k}^{2}\rangle =\frac{2k_{B}T}{\pi r_{\mathrm{av}}\gamma }\left(\frac{1}{k^{2}-1}\right)$$

where brackets <…> mean the average value of 30 images. The experimental data was fit with Equation (4) and we obtained the line tension γ. We measured the line tension of five to 10 domains for each condition.

#### 2.2.3. DSC Measurements

DPPC, Chol, and the LAs were dissolved in chloroform, at concentrations of 300, 150 and 150 mM, respectively, and were subsequently stored at −20 °C. The solutions were transferred into test tubes, and the desired composition was mixed to give a total volume of 60 μL. The mixed solutions were dried under a gentle stream of nitrogen gas, and formed into lipid films. To evaporate all of the remaining solvent, samples were dried under vacuum for at least 3 h. Lipid films were hydrated with Milli-Q water (60 μL), and sonicated for 1 h at least once at 50–60 °C to allow them to be peeled from the bottom of the test tube. The final concentration of the lipid/LA mixtures in liposomal solution was 150 mM. The compositions examined were DPPC/LAs 100:0, 97.5:2.5, 95:5, 92.5:7.5, and 90:10; and DPPC/Chol/LAs 90:10:0, 87.75:9.75:2.5, 85.5:9.5:5, 83.25:9.25:7.5, and 81:9:10.

Thermographs were obtained by a DSC-822e (Mettler Toledo International Inc., Greifensee, Switzerland). A total of 12–15 μL of liposomal solutions were placed into aluminum sample pans after stirring by a vortex mixer for over 30 s. We used sample pans filled with Milli-Q water as reference cells. The weight of the water was identical in both the reference and sample cells. Heating/cooling cycles were performed three times between 20 and 60 °C, and the third heating process is shown as a representative thermograph. Heating and cooling rates were set at 5 °C/min. Each heating/cooling scan was started after a 3–5 min pre-scan incubation at 20/60 °C. All raw data were normalized by the weights of the samples. The same measurements were performed at least three times to ensure reproducibility of the data.

**Figure 2:**
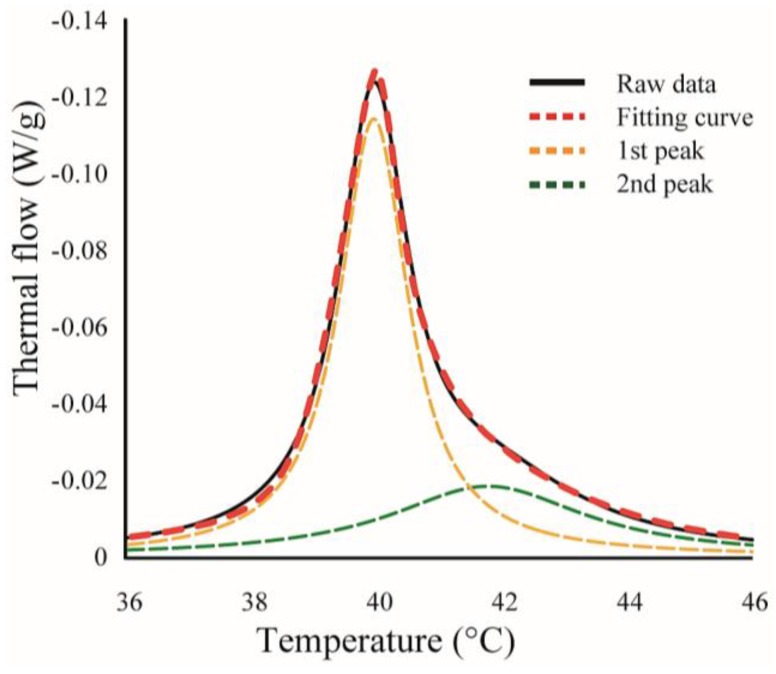
An example for curve fitting. Black-solid and red-dashed lines indicate experimental data and calculated fitting curve, respectively. Yellow- and green-dashed lines are the curves for two independent transitions, and the sum of these curves corresponds to the calculated fitting curve (red-dashed line).

We found some asymmetrical peaks at several compositions and they could be described as a linear combination of two independent transitions. An asymmetrical peak is expressed as a linear combination between two Lorentzian functions, as shown in Figure 2.

## 3. Results

### 3.1. Miscibility Temperature Measurement in LA-Containing Lipid Membranes

**Figure 3:**
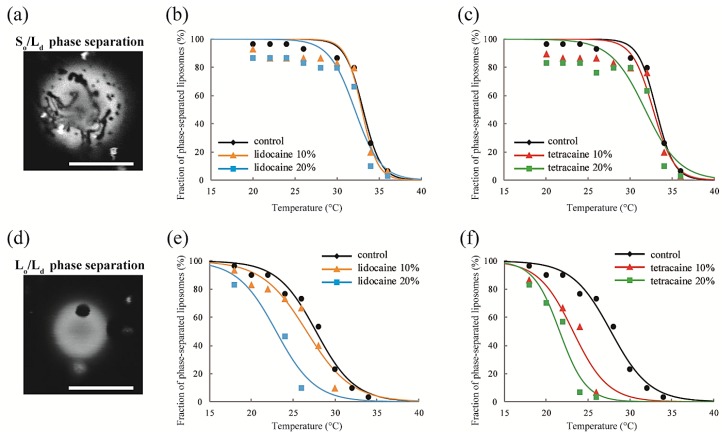
(**a**) Microscopy image of S_o_/L_d_ phase-separated liposomes. Scale bar 10 μm; (**b**,**c**) The fractions of phase-separated liposomes as a function of temperature in DOPC/DPPC/lidocaine and DOPC/DPPC/tetracaine, respectively. We fixed the DOPC:DPPC ratio to 1:1; (**d**) Microscopy image of L_o_/L_d_ phase-separated liposomes. Scale bar 10 μm; (**e**,**f**) The fractions of phase-separated liposomes as a function of temperature in DOPC/DPPC/Chol/lidocaine and DOPC/DPPC/Chol/tetracaine, respectively. We fixed the DOPC:DPPC:Chol ratio to 2:2:1.

We observed the effects of LAs on the thermostability of phase-separated structures in multicomponent lipid membranes. First, we investigated the lipid mixtures consisting of DOPC/DPPC/LAs without Chol. In this experiment, we fixed the ratio of DOPC/DPPC to 1:1 and set the LA concentrations to 0%, 10%, and 20%. At lower temperatures, coexistence between the DOPC-rich L_d_ phase and DPPC-rich S_o_ phase was observed, as shown in Figure 3a. Rhod-DHPE is localized in the L_d_ phase; the bright and dark regions correspond to the L_d_ and S_o_ phases, respectively. The anisotropic shape domains can be regarded as S_o_ domains. To examine the thermostability of phase-separated domains, we measured the fraction of phase-separated liposomes as the temperature increased. The results following the addition of lidocaine or tetracaine are shown in Figure 3b,c, respectively. In all cases, the fractions of the phase-separated liposomes decreased as the temperature increased. This is because the mixing entropy becomes predominant at higher temperatures. The temperature at which the fraction of phase-separated liposomes reaches 50% is the miscibility temperature (*T*_mix_). To identify the miscibility temperature of each lipid mixture, we fit the experimental data with Equation (1). The obtained miscibility temperatures were *T*_mix_ = 33.1 °C (control), 32.9 °C (lidocaine 10%), 32.1 °C (lidocaine 20%), 32.7 °C (tetracaine 10%), and 31.8 °C (tetracaine 20%). We did not observe any significant changes in the miscibility temperatures after adding LAs. Therefore, S_o_/L_d_ phase separation was not significantly affected by the LAs.

Next, DOPC/DPPC/Chol/LAs lipid mixtures were investigated to gain an understanding of the effects of Chol and LAs on phase separation. We fixed the DOPC/DPPC/Chol ratio to 2:2:1 and set the LA concentrations to 0%, 10%, and 20%. In the presence of Chol, the S_o_ phase transforms into the L_o_ phase [17]. Therefore, L_o_/L_d_ phase separation is observed in most of the liposomes at lower temperatures, as shown in Figure 3d. Here, the bright and dark regions correspond to the L_d_ and L_o_ phases, respectively. The miscibility temperatures were *T*_mix_ = 27.7 °C (control), 26.6 °C (lidocaine 10%), 23.0 °C (lidocaine 20%), 23.2 °C (tetracaine 10%), and 21.6 °C (tetracaine 20%). Although we did not find any significant changes in the miscibility temperatures in DOPC/DPPC/LA systems without Chol, they clearly decreased as the LA concentrations increased in lipid mixtures with Chol. The reduction in miscibility temperature with tetracaine was greater than that with lidocaine.

### 3.2. Line Tension Measurement at the Liquid Domain Boundary in LA-Containing Lipid Membranes

**Figure 4:**
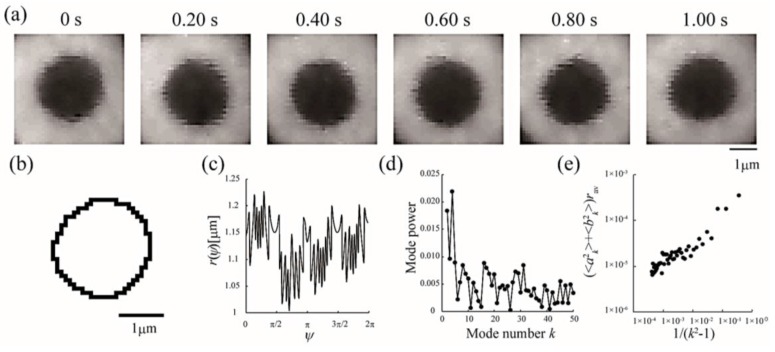
Analytical sequences of line tension at the L_o_/L_d_ phase boundary from domain boundary fluctuations. (**a**) Sequential images of a fluctuating L_o_ domain at a DOPC/DPPC/Chol ratio of 40:40:20 membrane at 28 °C; (**b**) Domain boundary trace of the image in (**a**) at 0 s; (**c**) Radial fluctuation as a function of the polar angle ψ (°); (**d**) Power spectrum calculated from (**c**); (**e**) Average Fourier coefficients obtained from 30 images plotted against 1/(*k*^2^ − 1).

Line tension at the domain boundary is one of the most important factors affecting the stability of phase-separated domains. Generally, the line tension at the L_o_ domain boundary is about ~3 pN [31,32]. Here, we obtained the line tension at the L_o_ domain boundary in LA-containing lipid membranes by the flicker spectroscopy of domain boundary fluctuations. We analyzed several images in which an isolated domain was present at the center of the liposome surface. Because this analysis calculates displacement from the circular shape, we ignored the non-circular domain shown in Figure 3a. The domain boundary fluctuation was imaged for 1 s at 30 frames/s (Figure 4a) and the trace of the domain boundary was obtained by ImageJ (Figure 4b). The radial fluctuation is plotted in Figure 4c and the power spectrum calculated from the radial fluctuation is shown in Figure 4d. The average Fourier coefficients obtained from 30 images are plotted in Figure 4e, and the line tension was calculated from the slope of the plot by Equation (4).

**Figure 5:**
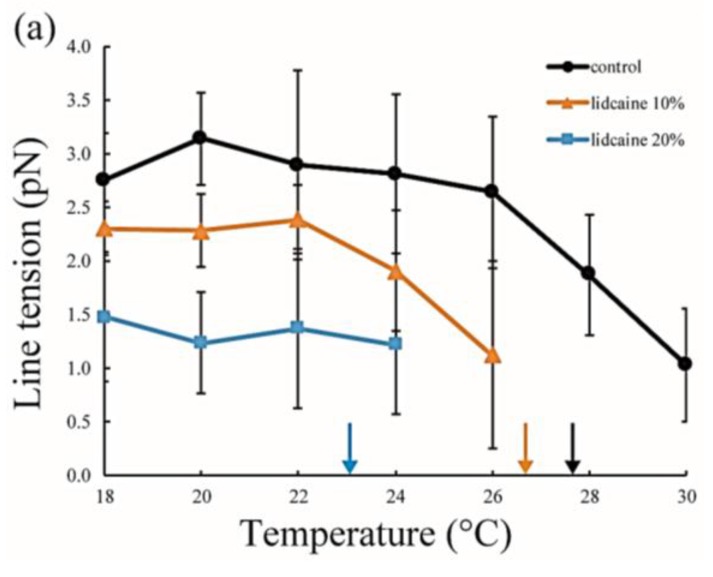

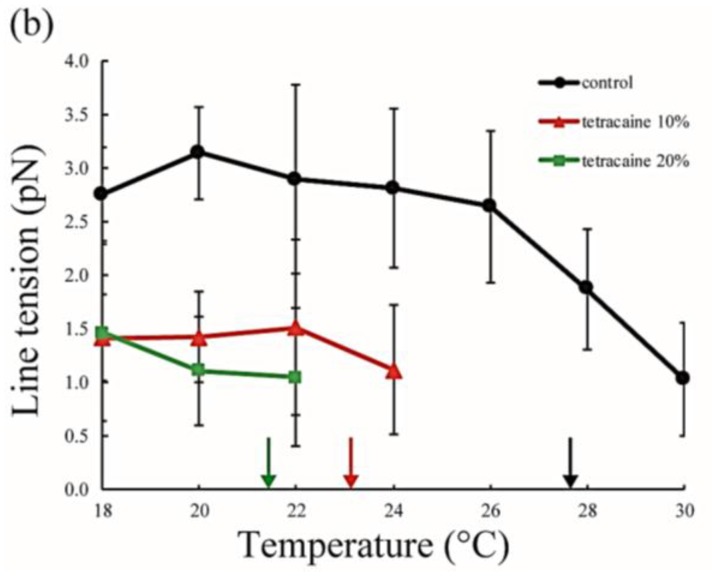
Line tension at the L_o_/L_d_ phase boundary in DOPC/DPPC/Chol/LA mixtures as a function of temperature. Black lines with circles indicate the control (DOPC/DPPC/Chol mixture without LAs), and colored lines with triangles and squares show LAs at 10 and 20 mol % added to the membranes, respectively. Orange and blue lines denote lidocaine-added membranes (**a**); and red and green lines denote tetracaine-added membranes (**b**); Miscibility temperatures measured by microscopic observations in Figure 3 are shown as arrows. Colors of the arrows correspond to those of the lipid compositions.

The line tension values are summarized in Figure 5. Without LAs, the line tension was about 3 pN at room temperature (20–22 °C), and this value is in good agreement with that reported in previous studies [31,32]. As the temperature increases, the line tension decreases just before reaching the miscibility temperature. When the temperature approaches the miscibility temperature, the composition difference between the coexisting two phases generally becomes smaller. Therefore, the line tension is reduced because the differences in physical properties between the two phases (spontaneous curvature, membrane thickness, and chain ordering) become smaller. We saw the same tendency in our systems containing LAs. Specifically, the line tension in the system containing LAs was lower than that in the system without LAs. Moreover, it decreased in an LA-concentration manner. The reduction in line tension of the tetracaine-containing membranes was larger than that of lidocaine-containing membranes. This tendency was largely consistent with the results obtained in our miscibility temperature measurements, and clearly shows that LAs strongly decrease the line tension at the L_o_/L_d_ domain boundary. The previous study also indicated that the line tension in a DOPC/DPPC/Chol mixture is quantitatively decreased by the addition of dibucaine, which is one of the LAs [33]. We explain why LAs reduce the line tension in the Discussion.

### 3.3. DSC Measurement in LA-Containing Lipid Membranes

**Figure 6:**
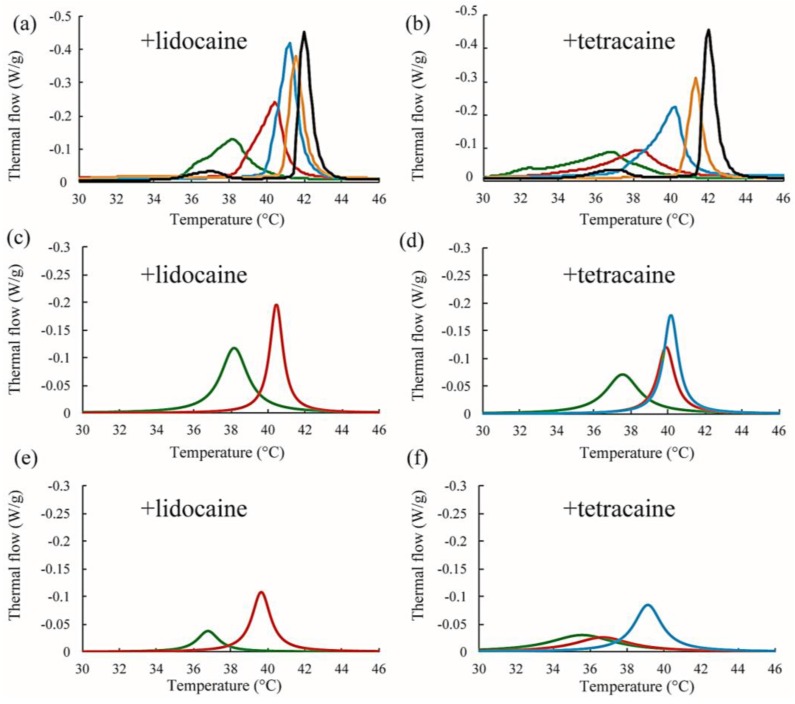
(**a**,**b**) Representative DSC thermographs of DPPC/lidocaine and DPPC/tetracaine membranes, respectively. Black, orange, blue, red, and green lines indicate LA at 0, 2.5, 5, 7.5, and 10 mol %, respectively; (**c**,**d**) Stronger peaks obtained from peak deconvolution in the DPPC/lidocaine and DPPC/tetracaine membranes, respectively; (**e**,**f**) Weaker peaks obtained from peak deconvolution in DPPC/lidocaine and DPPC/tetracaine membranes, respectively. Since the peak shape is almost symmetrical for lidocaine below 5 mol % and tetracaine below 2.5 mol %, we did not perform peak deconvolution in (**c**–**f**).

Next, we discuss the effects of LAs on the thermostability of lipid membranes based on DSC. The representative thermographs for DPPC/LA mixtures are shown in Figure 6. Sharp peaks can be seen at 42 °C that correspond to the main transition of the lipids between solid (ripple) phase and liquid phase, and the broad peak at 37 °C corresponds to the pretransition state between the solid and ripple phases in the DPPC single component system without LAs, shown as black lines in Figure 6a,b. This thermograph is consistent with some previous reports [17,21,22]. Since the pretransition peak disappears as the LA concentration increases, we focused on the main transition peak. When both lidocaine and tetracaine were present, the main transition peak shifted toward a lower temperature as the LA concentration increased. Interestingly, the shape of the peak became asymmetrical at a lidocaine concentration of 7.5% and tetracaine concentration of 5%. We presumed that the asymmetrical peak could be described as a linear combination of two independent transitions. The results of peak deconvolution are shown in Figure 6c–f. We expressed an asymmetrical peak as a linear combination between two Lorentzian functions. Figure 5c,d show stronger peaks at higher temperatures, corresponding to the transitions of the DPPC-rich phase. On the other hand, the weaker peaks at lower temperatures, shown in Figure 6e,f, correspond to the transition of the LA-rich phase. Therefore, as the concentration of the LA increased, some molecules could not dissolve in the DPPC membranes, which led to the formation of an LA-rich phase. This result implies a low affinity between DPPC and LAs. Moreover, compared with lidocaine, tetracaine seemed to show a lower affinity to DPPC.

**Figure 7:**
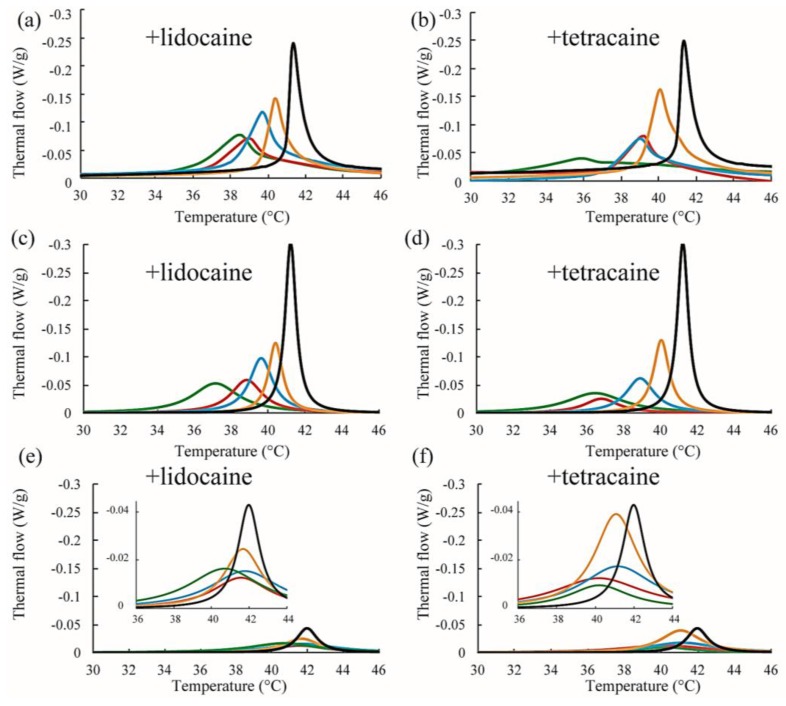
(**a**,**b**) Representative DSC thermographs of DPPC/Chol/lidocaine and DPPC/Chol/tetracaine membranes, respectively. Black, orange, blue, red, and green lines indicate LA at 0, 2.5, 5, 7.5, and 10 mol %, respectively; (**c**,**d**) Stronger peaks obtained from peak deconvolution in DPPC/Chol/lidocaine and DPPC/Chol/tetracaine membranes, respectively; (**e**,**f**) Weaker peaks obtained from peak deconvolution in DPPC/Chol/lidocaine and DPPC/Chol/tetracaine membranes, respectively. Insets show magnifications of the peaks.

**Figure 8:**
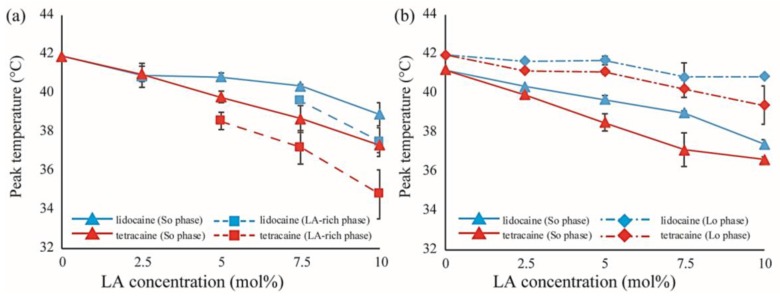
(**a**) Shifts in peak temperatures obtained from peak deconvolution of the DSC thermographs in DPPC/LA mixtures as a function of LA concentration. Blue and red lines represent lidocaine and tetracaine-containing membranes, respectively. Solid and dashed lines indicate the temperature shifts of the stronger peak (S_o_ phase) and weaker peak (LA-rich phase), respectively; (**b**) Shifts in peak temperatures obtained from peak deconvolution of the DSC thermographs in DPPC/Chol/LA mixtures as a function of LA concentration. Blue and red lines represent lidocaine and tetracaine-containing membranes, respectively. Solid and dot-dashed lines indicate temperature shifts of the stronger peak (S_o_ phase) and weaker peak (L_o_ phase), respectively.

We also performed DSC measurements for the mixtures of DPPC/Chol/LAs. The thermograph of the DPPC/Chol binary mixture without an LA is denoted by a black line in Figure 7a,b. A clear peak can be seen around 41.5 °C. It has been reported that phase separation between the S_o_ and L_o_ phases occurs at a DPPC/Chol ratio of 90:10 [34]. As per the DPPC/LA mixtures, we performed peak deconvolution based on a two-state transition; the strong and weak components are shown in Figure 7c–f, respectively. These strong and weak peaks correspond to the DPPC-rich S_o_ and Chol-rich L_o_ phases, respectively [34]. In Figure 7c,d, the stronger peaks shift toward a lower temperature as the LA concentration increases. This behavior resembles that of DPPC-rich phase in DPPC/LA binary mixtures, as indicated in Figure 6. On the other hand, the positions of the weaker peaks were not significantly changed by the addition of LAs, as shown in Figure 7e,f. In Figure 8, we summarized the temperature shifts of both the strong and weak peaks. The temperature shift of the S_o_ phase in the DPPC/LA mixtures was almost identical to that of the S_o_ phase in the DPPC/Chol/LA mixtures. Since an LA-rich phase is formed in DPPC/LA mixtures, the S_o_ phase in DPPC/LAs can be considered an LA-poor phase. Therefore, we speculate that the S_o_ phase in DPPC/Chol/LA mixtures also becomes an LA-poor phase. On the other hand, the clear LA-rich phase was not found in the DPPC/Chol/LA mixtures. In DPPC/Chol/LAs systems, we believe that large amounts of LAs are included in the L_o_ phase. We further discuss LA localization in heterogeneous membranes based on the DSC results in Section 4.

## 4. Discussion

Microscopic observation revealed that the miscibility temperature (*T*_mix_) of the phase-separated structures on DOPC/DPPC membranes was not significantly changed by adding LAs (Figure 3b,c). Furthermore, according to the results of the DSC experiment on DPPC/LA mixtures, an LA-rich phase was formed at higher concentrations of LAs (Figure 6a,b), which suggests a low affinity between DPPC and LAs. Therefore, most LA molecules can be partitioned into DOPC-rich L_d_ phases in DOPC/DPPC/LAs membranes. LAs did not impart any crucial effects on the DPPC-rich S_o_ phase, and the miscibility temperature in DOPC/DPPC/LA mixtures was not shifted by them.

On the other hand, as the concentrations of LAs increased, the miscibility temperatures for DOPC/DPPC/Chol/LA lipid membranes decreased and the thermostability of the L_o_ phase was lowered (Figure 3e,f). From the results of the DSC experiments, the shift in the peak temperature of the S_o_ phase in the DPPC/LA mixtures was almost identical to that of the S_o_ phase in the DPPC/Chol/LA mixtures (Figure 8). Therefore, we can consider that the amount of LA in both S_o_ phases was almost the same. The DSC results for the DPPC/LA mixtures indicated that the S_o_ phase corresponded to the LA-poor phase. Based on this finding, the S_o_ phase for DPPC/Chol/LA mixtures can be also regarded as the LA-poor phase. In addition, we could not find a clear LA-rich phase among the DPPC/Chol/LA mixtures. Therefore, a large amount of LA molecules may be included in the L_o_ phase in these lipid mixtures. This implies that the presence of Chol promotes the partitioning of LAs. Although a large amount of LA may have been partitioned into the DOPC-rich L_d_ phase in DOPC/DPPC/Chol/LA membranes, some was included in the L_o_ phase. The LA molecules in the L_o_ phase significantly decreased the thermostability. We can think of two mechanisms that explain why the presence of Chol enhances the partitioning of LAs into L_o_ phase. First, the DPPC-rich phase transitions to the liquid phase from the solid phase in the presence of Chol. Therefore, LA can be partitioned into the L_o_ phase, because Chol loosens the packing between the DPPC molecules. This hypothesis indicates that Chol indirectly promotes the partitioning of LAs into L_o_ phase. The second hypothesis is a direct attraction between LAs and Chol. Because LAs and Chol are hydrophobic molecules, they are buried in the hydrophobic region of lipid membranes and may directly interact with each other. This mechanism could be revealed by a future NMR study.

The distribution of LAs into the L_o_ phase is also supported by the line tension measurements. The LAs decreased the line tension at the L_o_/L_d_ domain boundary in DOPC/DPPC/Chol membranes (Figure 5). Some studies have mentioned that the additive molecules adsorb onto the domain interface and reduce the line tension [35,36,37,38,39,40]. Such molecules are called “linactant” based on an analogy to a surfactant [38]. Although the line energy at the domain boundary is decreased by the adsorption of linactants, entropic loss arises instead of line energy gain. Therefore, it is difficult for a molecule to be a linactant, because it would have to possess a structure with an affinity for both coexisting phases. Since the LAs used in this study do not seem to have such structures (see Figure 1), they may not be linactants. Therefore, we think that some LAs are incorporated within the L_o_ phase, as mentioned above. As a result, the chain ordering of the L_o_ phase is disturbed by the presence of LAs. The differences in physical properties between the two coexisting phases (spontaneous curvature, membrane thickness, chain ordering) become smaller, which decreases the line tension. This mechanism was indicated by electron spin resonance [41], and Laurdan generalized the polarization measurements among different lipid mixtures [42]. On the other hand, according to our DSC experiments, the transition temperatures of lipids in the L_o_ phase were not significantly changed by adding LAs. A similar tendency has been reported in some other studies [24,27]. However, it is important to investigate the relationship between chain ordering and the transition temperature in future studies.

Tetracaine showed more significant effects than lidocaine on the physical properties of the lipid membranes, such as the miscibility temperature, line tension at the L_o_/L_d_ interface, and main transition temperature. These findings are consistent with the results of a previous study, which reported that tetracaine suppresses phase separation at room temperature more strongly than lidocaine [25]. Moreover, Paiva et al. showed that tetracaine has a stronger effect than lidocaine on the transition temperature of raft-mimetic membranes [23]. Generally, tetracaine is a more powerful and toxic LA than lidocaine [43]. In other words, tetracaine shows anesthetic and cytotoxic effects at lower concentrations than lidocaine. Thus, from these results, the potencies of the LAs and suppression of phase separation may be associated. Furthermore, *n*-alcohol general anesthetics are reported to correlate with pharmacological strength and membrane phase behavior [28]. As the length of the hydrocarbon tail of alcohol becomes longer, the lipid transition temperature decreases and the strength of the anesthesia becomes stronger. Therefore, increased hydrophobicity leads to increased anesthetic action. Moreover, an LA oil/water partitioning experiment showed that the action of the anesthetic and the molecular hydrophobicity are correlated [44]. On the other hand, we suggested the importance of the affinity between the L_o_ phase and LAs. Thus, the relationship between the molecular hydrophobicity of LAs and anesthetic action based on the stability of the L_o_ phase on the addition of LAs should be evaluated in future studies by an NMR investigation of the interactions between LAs and the L_o_ phase.

Since a large part of LA is hydrophobic, we discussed the phase behavior based on the hydrophobic interaction between lipid membranes and LAs. LAs are medically used as chloride salts to increase the water solubility. Although LAs were directly included in lipid membranes in our study, LAs were added to lipid membranes by dissolving them in water phase in some studies [33]. However, because the experimental results about the main transition temperature shift and phase behavior are almost consistent with our study, the behavior of charged LAs is similar to that of uncharged LAs. Therefore, we believe that the hydrophobic interaction between LAs and lipid membranes is the most important factor. Moreover, in actual living cells, the electric charges on LAs are well screened by many kinds of cations in the vicinity of cell membranes, and the effect of electric charges on LAs may be negligible. On the other hand, some studies mentioned the importance of electric charges on the phase separation in multicomponent lipid membranes [45]. The studies on the hydrogen bonding between LAs and phospholipids, and the effect of LAs on the hydration water near the surface of an LA-containing lipid membrane, as well as the electric charge of LAs, will be important in the near future.

We showed that the raft-mimicking structure (L_o_ phase) is destabilized by adding LAs. If raft domains are also destabilized by LAs in the cell membranes, the sodium ion channel proteins present may be strongly affected. Voltage-dependent sodium channels form gate structures that allow the selective permeation of specific cations by subunits in the structure-forming multimers [46]. The gate structure is controlled by miniscule structural changes in the proteins of well organized subunits. If the raft structure is altered by LAs, the order, structure, and movement of the subunits forming the gate structure may be impaired. In fact, Brohawn et al. found that the function of ion channels placed in membranes isolated from cells can be controlled by the external pressure achieved by poking the membranes with a glass capillary [12]. The application of external pressure to membranes is known to change the phase behavior [47] and impact the anesthetic dose. Therefore, the membrane proteins in raft domains are influenced by changes in the physical properties of the membranes, such as rigidity, thickness, fluidity, and phase. Future studies should investigate the mechanisms controlling channel activities via changes in the phase state of the membranes.

## 5. Conclusions

In this study, we demonstrated the changes mediated by the thermal behavior of phase-separated structures in the presence of LAs. LAs lowered the miscibility temperature (*T*_mix_) in DOPC/DPPC/Chol/LA mixtures, whereas the miscibility temperature of DOPC/DPPC/LA mixtures without Chol did not change significantly. Moreover, we also showed that the line tension at the L_o_/L_d_ domain boundary was reduced by LAs. DSC measurements revealed that LAs could be partitioned into the L_o_ phase with the help of Chol. Subsequently, the miscibility temperature and the line tension are decreased significantly.

Voltage-dependent sodium ion channels, which could be deactivated by LAs, are known to exist on lipid rafts in the cellular membrane. Our results suggest that LAs destabilized raft-mimetic structures composed of saturated phospholipids and Chol. The change in phase behavior could influence the gating or binding abilities of membrane proteins, such as ion channels. Based on our results, the relationship between ion channel activity and the stability of raft domains should be further investigated.
